# Supplementary material for: Complex‐centric proteome profiling by SEC‐SWATH‐MS
Source: Mol Syst Biol. 2019 Jan 14;15(1):e8438. doi: 10.15252/msb.20188438 (PMC6346213; doi:10.15252/msb.20188438)
Supplement: Supplementary file 8 — Dataset EV7 [file MSB-15-e8438-s008.zip › feature_plots_string/Q9UJX5.pdf]

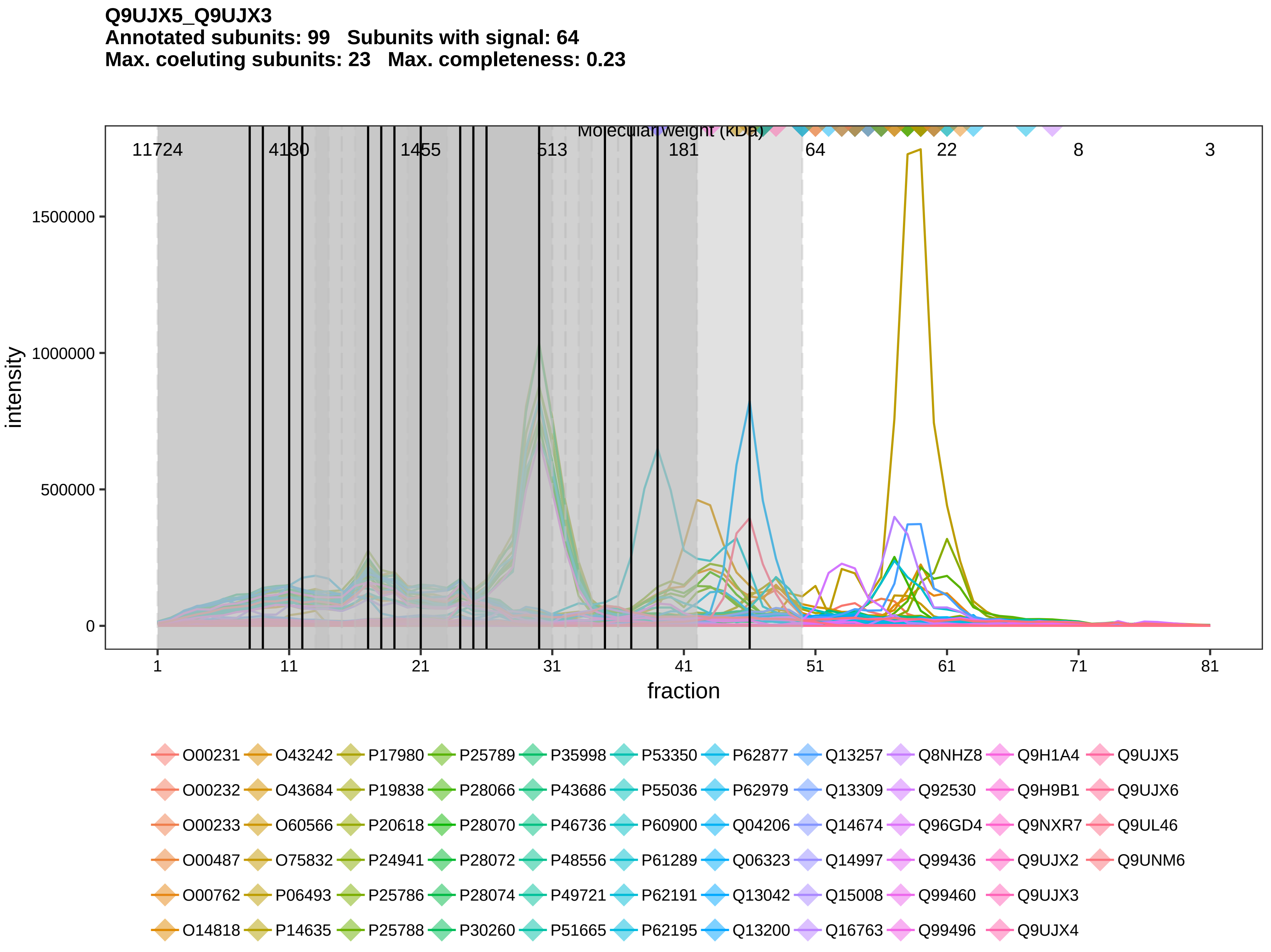
Q9UJX5_Q9UJX3
Annotated subunits: 99 Subunits with signal: 64
Max. coeluting subunits: 23 Max. completeness: 0.23
Molecular weight (kDa)
11724
4130
1455
513
181
64
22
8
3
intensity
1500000
1000000
500000
0
fraction
1
11
21
31
41
51
61
71
81
O00231
O00232
O00233
O00487
O00762
O14818
O43242
O43684
O60566
O75832
P06493
P14635
P17980
P19838
P20618
P24941
P25786
P25788
P25789
P28066
P28070
P28072
P28074
P30260
P35998
P43686
P46736
P48556
P49721
P51665
P53350
P55036
P60900
P61289
P62191
P62195
P62877
P62979
Q04206
Q06323
Q13042
Q13200
Q13257
Q13309
Q14674
Q14997
Q15008
Q16763
Q8NHZ8
Q92530
Q96GD4
Q99436
Q99460
Q99496
Q9H1A4
Q9H9B1
Q9NXR7
Q9UJX2
Q9UJX3
Q9UJX4
Q9UJX5
Q9UJX6
Q9UL46
Q9UNM6
